# Amygdala Plasticity and Pain

**DOI:** 10.1155/2017/8296501

**Published:** 2017-12-10

**Authors:** Jeremy M. Thompson, Volker Neugebauer

**Affiliations:** ^1^Department of Pharmacology and Neuroscience, Texas Tech University Health Sciences Center, School of Medicine, Lubbock, TX, USA; ^2^Center of Excellence for Translational Neuroscience and Therapeutics, Texas Tech University Health Sciences Center, Lubbock, TX, USA

## Abstract

The amygdala is a limbic brain region that plays a key role in emotional processing, neuropsychiatric disorders, and the emotional-affective dimension of pain. Preclinical and clinical studies have identified amygdala hyperactivity as well as impairment of cortical control mechanisms in pain states. Hyperactivity of basolateral amygdala (BLA) neurons generates enhanced feedforward inhibition and deactivation of the medial prefrontal cortex (mPFC), resulting in pain-related cognitive deficits. The mPFC sends excitatory projections to GABAergic neurons in the intercalated cell mass (ITC) in the amygdala, which project to the laterocapsular division of the central nucleus of the amygdala (CeLC; output nucleus) and serve gating functions for amygdala output. Impairment of these cortical control mechanisms allows the development of amygdala pain plasticity. Mechanisms of abnormal amygdala activity in pain with particular focus on loss of cortical control mechanisms as well as new strategies to correct pain-related amygdala dysfunction will be discussed in the present review.

## 1. The Amygdala and Pain

The amygdala is an almond-shaped limbic structure located in the medial temporal lobe and is well known for its role in conveying emotional significance to a sensory stimulus, emotional and affective states, and related behavioral adaptations in response to changes in the internal and external bodily environment [1–4]. The amygdala has also emerged as an important site in the brain for the emotional-affective dimension of pain and pain modulation [5–12].

A pain-related function was first suggested by the discovery of a dedicated nociceptive pathway from the spinal cord through the external lateral parabrachial (PB) nucleus to the central nucleus of the amygdala [13, 14]. Reevaluation of an historical example of reduced pain sensitivity also suggests amygdala involvement in pain processing. Patient H.M. was a man that underwent bilateral resection of the temporal lobe including the uncus, amygdala, anterior hippocampus, and parahippocampal gyrus to correct severe and intractable epilepsy [15–17]. After the surgery, H.M. did not perceive even the highest thermal stimulus intensity as painful when control groups did. It is now thought that this deficit was likely due to amygdala resection [16, 17], illustrating the importance of the amygdala in pain processing in the brain. Importantly, this deficit in pain perception occurred despite an intact nociceptive system and was not accompanied by the tissue injury characteristic of pain insensitivity disorders, indicating that protective pain functions were intact.

Since the initial discovery of nociceptive pathways to the amygdala, preclinical [5, 7, 8] and clinical [10, 11, 18, 19] studies have provided direct support for amygdala involvement in pain. Electrophysiological recordings in anesthetized rats in vivo and in rodent brain slices in vitro and molecular biological assays showed increased activity markers in response to acute noxious stimuli, including mechanical or thermal stimulation [20, 21], as well as in models of visceral pain [22–28], intraplantar formalin [29–31], acid-induced muscle pain [32], kaolin/carrageenan-induced monoarthritis [33–41], and chronic neuropathic pain [42–44].

The clinical relevance of these findings has been corroborated by human neuroimaging studies that demonstrate amygdala activation in response to experimental noxious stimuli, including mechanical compression, thermal stimulation, and capsaicin application [10], as well as increased amygdala activity in migraineurs compared to healthy controls when presented with negative but not positive or neutral emotional stimuli [45]. In addition, functional connectivity between the left amygdala and the PFC, cingulate cortex, and basal ganglia is different in patients with complex regional pain syndrome (CRPS) [46], and corticolimbic reverberating loops have been implicated in the prediction of and transitioning to chronic pain [11]. Patients with irritable bowel syndrome (IBS) had higher positive resting-state functional connectivity between the amygdala and the insula, pre- and postcentral gyri, and supplementary motor area compared to healthy controls, and this increased connectivity positively correlated to pain intensity [47]. A separate study demonstrated that IBS patients that did not have visceral hypersensitivity had decreased positive resting-state functional connectivity of the amygdala within the default mode network compared to healthy controls as well as IBS patients with visceral hypersensitivity [48]. In female twin pairs with and without chronic pelvic pain, connectivity between the right PAG and the right amygdala, connectivity between the left PAG and the right and left basolateral amygdala, and connectivity of the right basolateral amygdala to the medial orbital frontal cortex, anterior cingulate cortex (ACC), right insula, left thalamus, and hypothalamus differed between the twin with pelvic pain compared to the healthy twin before and after bladder distension by an oral water bolus [49].

## 2. Amygdala Pain Neurocircuitry

**Figure 1:**
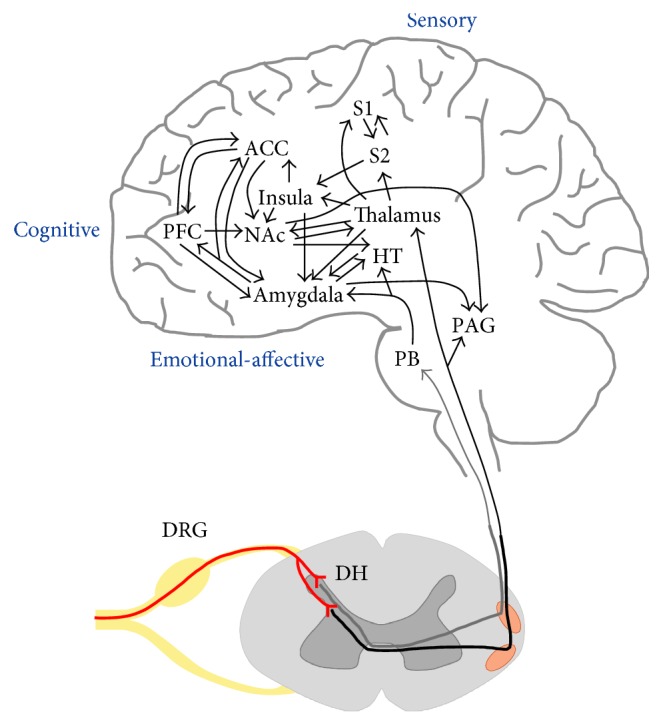
Pain neurocircuitry. Peripheral nociceptive afferent fibers (red lines) form synapses in the dorsal horn of the spinal cord. Axons of spinal dorsal horn neurons decussate in the anterior white commissure and travel in the ventrolateral funiculus (spinothalamic tract; black line) or the dorsolateral funiculus (spino-parabrachio-amygdaloid tract; gray line) to different targets in the brain. Sensory discriminative aspects of pain involve projections from the thalamus to somatosensory cortical areas. Cognitive aspects of pain involve integration within limbic and (prefrontal) cortical regions. Emotional-affective aspects of pain involve integrative processing in the limbic brain regions centered on the amygdala which is a key node. Circuitry is based on [6, 7, 15, 50–58]. Abbreviations: ACC, anterior cingulate cortex; DH, dorsal horn; DRG, dorsal root ganglion; HT, hypothalamus; NAc, nucleus accumbens; PAG, periaqueductal gray; PB, parabrachial nucleus; PFC, prefrontal cortex; S1/2, primary/secondary somatosensory cortex.

**Figure 2:**
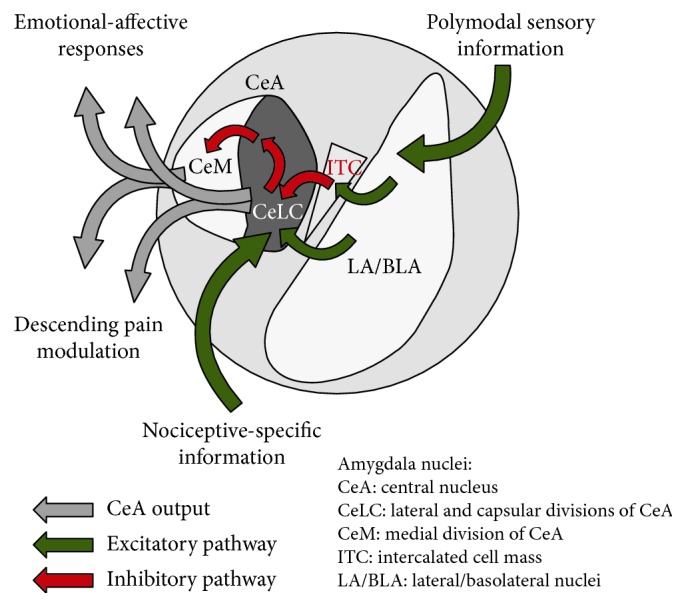
Amygdala pain neurocircuitry. Lateral-basolateral complex (LA/BLA), central nucleus (CeA), and intercalated cell mass (ITC) form the core circuitry involved in amygdala-dependent pain behaviors and pain modulation. The LA/BLA receives polymodal sensory information from cortical and thalamic areas and attaches emotional-affective information, which is then relayed to the CeA. This transmission includes direct excitatory projections to neurons in the lateral and capsular divisions of the CeA (CeLC) as well as feedforward inhibition of CeLC neurons through an LA/BLA projection to the ITC, a group of GABAergic interneurons between LA/BLA and CeA. ITC cells are also the target of cortical control from the mPFC. The CeLC integrates purely nociceptive information received via the spino-parabrachio-amygdaloid tract with highly processed information received from the LA/BLA to generate emotional-affective responses and contribute to top-down pain modulation via projections to the brainstem. This can be done through two types of amygdala outputs: one from CeLC projection neurons and the other from CeM neurons that can be disinhibited by CeLC neurons.

The amygdala receives multiple lines of input (Figure 1) relevant for pain processing, and multiple nuclei in the amygdala are involved in its pain processing functions. These include the lateral-basolateral complex (LA/BLA), the central nucleus (CeA), and the intercalated cell mass (ITC); see Figure 2 and [7–9].

The LA/BLA is predominantly composed of pyramidal glutamatergic projection neurons that receive polymodal sensory, including nociceptive, inputs from the midline and posterior nuclei of the thalamus, insular cortex, and sensory association cortices, as well as inputs from the ACC and medial prefrontal cortex (mPFC) [1, 7–9, 12]. Through associative processing, the LA/BLA attaches emotional-affective content to the sensory inputs and transmits that highly processed information to the amygdala output region in the CeA for further processing as part of amygdala fear and anxiety circuitry [3, 50, 59, 60]. This LA/BLA-CeA projection is now known to generate and modulate pain-related behaviors [7]. The BLA also projects to different cortical areas, including the infra- and prelimbic mPFC, ACC, and perirhinal and insular cortices [51, 61–67]. The BLA-mPFC projection is thought to provide emotional information for value-based executive functions [67–70] and has been implicated in pain-related cortical deactivation and cognitive control [6, 7, 11, 71].

The CeA serves as the major output nucleus for amygdala-driven pain-related functions. The laterocapsular region of the CeA (CeLC, including both the lateral and capsular divisions) receives purely nociceptive information via the spino-parabrachio-amygdaloid tract [72] and possibly via direct projections from the spinal cord [73, 74]. The parabrachial input is characterized by its peptidergic nature and serves as the exclusive source of calcitonin gene-related peptide (CGRP) for the amygdala [75]. The CeLC contains GABAergic projection neurons that also contain peptides such as corticotropin-releasing factor (CRF); they are characterized by their nonaccommodating spike firing pattern [7, 38, 76]. Nearly half of the CRF-CeA neurons receive CGRP input from the PB [77]. This nociceptive information is integrated with polymodal sensory information from the LA/BLA to generate amygdala-mediated responses important for pain behaviors and pain modulation [1, 7–9, 12].

Interposed between LA/BLA and CeLC is a group of GABAergic interneurons in the intercalated cell mass (ITC). ITC cells receive excitatory inputs from the infralimbic mPFC and LA/BLA and are activated during extinction of negative emotional responses [2, 3, 50, 78–82]. Therefore, ITC cells serve a gating function for amygdala output from the CeA through feedforward inhibition that involves activation of neuropeptide S (NPS) receptors on ITC cells [39, 83, 84].

CeA output arises from medial CeA (CeM) and CRF-CeLC projection neurons. These projections target other limbic, hypothalamic, and brainstem regions including the periaqueductal gray (PAG) [3, 60, 85–87]. Projections to hypothalamus and brainstem regions such as the nucleus of the solitary tract (NTS) contribute to amygdala-driven autonomic responses. Projections to the PAG generate or modulate vocalizations and startle responses. CeA projections to other limbic structures such as the bed nucleus of the stria terminalis (BNST) and to monoaminergic regions such as locus coeruleus are thought to drive amygdala influences over anxiety and depression [3, 88, 89]. CeLC neurons can also affect output from the CeM through intra-amygdala connections involving GABAergic protein kinase C delta (PKCδ) positive and other interneurons in the lateral CeA [50, 90–92], but details of this intra-amygdalar circuitry remain to be determined.

CeA neurons expressing PKCδ (PKCδ^+^) are inhibited by exposure to the fear-conditioned stimulus and have been termed “OFF cells,”, whereas PKCδ-negative neurons are excited when presented with the conditioned stimulus, and reciprocal inhibitory connections exist between these cell types [1, 90, 91]. Inhibition of PKCδ^+^ cells is believed to contribute to emotional responses such as freezing through disinhibition of CeM output neurons [50]. Importantly, PKCδ^+^ neurons are distinct from CRF-containing CeLC projection neurons characterized by their nonaccommodating spike firing pattern [87, 91]. And so, it is reasonable to assume that it is the population of PKCδ-negative CRF-CeLC neurons that have been studied for their nociceptive PB input and participation in pain processing [7]. CRF-CeLC neurons are uniquely positioned to provide and regulate amygdala output through their projections to extra-amygdalar targets and through local inhibition of PKCδ^+^ cells to disinhibit CeM output neurons [1, 3, 60, 85–87] (Figure 2).

## 3. Pain-Related Changes in Amygdala Neurocircuitry

Importantly, changes in the amygdala neurocircuitry have been detected in different preclinical models of pain and linked mechanistically to pain behaviors, indicating that these maladaptive neuroplastic changes are a brain mechanism of pain. In systems (whole animal) electrophysiology studies of amygdala neurons in anesthetized rats, background activity and responses to mechanical compression of peripheral tissues increased in an arthritis pain model in the CeLC [36, 37, 93–97] and BLA [71], as well as in the CeLC in a neuropathic pain model [42, 44]. These activity changes are not simply a reflection of changes along the pain pathways to the amygdala but arise from synaptic plasticity within the amygdala network. Brain slice physiology studies showed enhanced excitatory transmission at the PB-CeLC and BLA-CeLC synapses [33–35, 38, 40], decreased ITC-mediated synaptic inhibition of CeLC neurons [39, 40], and increased neuronal excitability in the CeLC [33, 34, 38], as well as enhanced excitatory transmission at the LA-BLA synapse and increased excitability of BLA neurons [71] in an arthritis pain model. Enhanced transmission at the PB-CeLC synapse and neuronal excitability [25] and increased levels of neurochemical activity markers (c-Fos and extracellular signal-regulated kinase, ERK) [22, 23, 28, 98] and CRF [24, 26] were found in the CeA in visceral pain models. Enhanced PB-CeLC and BLA-CeLC neurotransmission and excitability [43, 99] and increased expression of CRF and glucocorticoid receptors [100, 101] in the CeA were also observed in neuropathic pain models. It should be noted that not all of these neuroplastic changes have been described in all pain models. It is possible that amygdala neuroplasticity could differ based on time course (acute versus chronic pain) or etiology (inflammatory versus neuropathic) of pain. The full scope of pain-related amygdala plasticity remains an area of active investigation.

The clinical relevance of these neuroplastic changes in preclinical pain models is supported by neuroimaging studies, indicating that amygdala activity is increased in subjects with previously diagnosed pain conditions, including osteoarthritis, IBS, and fibromyalgia compared to matched controls [10].

Interestingly, this amygdala pain-related plasticity exhibits hemispheric lateralization. Under normal conditions, nociceptive inputs into the CeLC are received by and activate both the right and left amygdala [30, 36]. No apparent difference has been detected in background activity or responses to innocuous and noxious mechanical test stimuli between neurons in the right and left amygdala (CeLC), although neurons in the left amygdala appear to have more restricted receptive fields in peripheral tissues [36]. Exogenous activators also can increase neuronal activity in the right and left CeLC [36] or induce hypersensitivity [102]. In inflammatory [36] and neuropathic [42] pain conditions, neurons in the right but not left amygdala exhibit a sustained increase in background and evoked activity irrespective of the side of injury, and the receptive field size of neurons in the right but not left amygdala is increased in an arthritis pain model [36]. In humans, differences in connectivity between the right and left amygdala have been observed in IBS patients compared to healthy controls [47], IBS patients compared to those without visceral hypersensitivity [48], and twin pairs with one twin with and one without chronic pelvic pain before and after bladder distension [49].

Mechanisms of hemispheric lateralization of amygdala plasticity and function are not yet clear. Protein kinase A (PKA) activation has been implicated in the development of central sensitization [103] and synaptic plasticity [33–35, 104] in the CeLC in an arthritis pain model, and application of a PKA inhibitor into the right but not left amygdala resulted in a reduction in neuronal activity in this pain model [36], indicating that PKA is endogenously activated in the right but not left amygdala in the pain state. ERK is also known to play an important role in amygdala plasticity and amygdala-driven behaviors in inflammatory pain models [30, 104], and activation of the metabotropic glutamate receptor 5 (mGluR5) has been shown to increase amygdala ERK activation and ERK-dependent pain behaviors [102, 105]. Application of a blocker of ERK activation [30, 31] and pharmacological blockade or conditional deletion of mGluR5 [102] prevented formalin-induced hypersensitivity when administered into the right but not left amygdala. Chemical (pituitary adenylate cyclase-activating polypeptide) or optogenetic activation of the right but not left CeA increased nociceptive visceromotor responses in a murine urinary bladder distension model, and optogenetic silencing of the left but not right CeA increased bladder distension-induced visceromotor responses [27]. In a visceral pain model cyclophosphamide-induced cystitis, optogenetic activation of the left but not right CeA inhibited abdominal mechanosensitivity, whereas activation of the right CeA further increased visceromotor responses in this model [27] These findings may suggest that while neuroplasticity in the right CeA drives pain-related behaviors, the left CeA may be linked to antinociception.

Evidence from neuroimaging studies in humans also suggests right hemispheric lateralization of amygdala responses to acute experimental pain stimuli [10], whereas in clinical pain conditions, right hemispheric deactivation [106] and left hemispheric signal increases [10] have been reported. This could be due to a compensatory increase in inhibitory transmission in the left amygdala that is not present in the right amygdala in the chronic pain state. Pain-related hemispheric lateralization may reflect a general principle of lateralized emotional processing. Right hemispheric amygdala activation has been found in response to masked fearful faces [107] and was exaggerated in veterans with posttraumatic stress disorder (PTSD) [108]. Enhanced activity of the right, but not the left, amygdala in men was related to encoding and long-term memory of films judged as arousing negative emotions compared to neutral films [109, 110]. Mechanisms and significance of pain-related amygdala lateralization remain to be determined.

## 4. Pain-Related Amygdala-Centered Corticolimbic Interactions

Information processing in the amygdala can be regulated by inhibitory gating mechanisms centered on ITC cells and their activation by cortical control systems (Figure 2). The mPFC influences amygdala function through feedforward inhibition of CeLC neurons via excitatory projections to ITC cells as an important mechanism of cognitive modulation of emotions such as a fear [2, 3, 50, 78, 79, 81, 82, 88, 111]. Evidence suggests that mPFC-driven feedforward inhibition of CeLC output neurons is impaired in pain [7, 112]. Electrical stimulation of the external capsule, including infralimbic mPFC inputs into the amygdala, resulted in a non-*N*-methyl-D-aspartate (non-NMDA) receptor-mediated monosynaptic excitatory synaptic response (EPSC) in dorsomedial ITC cells and non-NMDA receptor-driven synaptic inhibition (IPSC) of CeLC neurons [39]. In brain slices from arthritic rats, the monosynaptic EPSC in ITC cells and the glutamate-driven IPSP in CeLC neurons were reduced [39], suggesting pain-related impairment of mPFC-driven feedforward inhibition of amygdala output.

Decreased infralimbic mPFC activity has been implicated in extinction deficits [113–116]. Accumulating evidence points to mPFC deactivation in pain, which could explain impaired control of amygdala processing [7, 112]. Functional and structural abnormalities in the mPFC have been detected in human pain patients [117, 118] and in preclinical pain models [71, 119–121]. As a consequence, activity of output neurons in the infralimbic and prelimbic mPFC is decreased in acute [71, 122, 123] and chronic pain models [120, 124, 125]. Decreased glutamatergic drive of pyramidal cells [120] and abnormally enhanced glutamatergic activation of parvalbumin-expressing GABAergic interneurons [71, 125, 126] have been implicated in the mPFC deactivation in pain.

Hyperactivity in the BLA plays an important role in pain-related mPFC deactivation [7, 112]. The BLA sends glutamatergic projections to the pre- and infralimbic mPFC [61, 66, 67]. Importantly, while some of these BLA axon terminals make direct contact with pyramidal cells, the majority of synapses on neighboring parvalbumin and somatostatin-positive interneurons form GABAergic connections with pyramidal cells, targeting mainly the somatic and proximal axonal regions [62, 127]. This synaptic arrangement was shown to account for amygdala-driven mPFC deactivation by glutamate-driven feedforward inhibition in an arthritis pain model [71, 126]. Feedforward inhibition involves activation of GABAergic interneurons mediated by non-NMDA receptors and mGluR1 but not mGluR5 [122, 128]. It should be noted that mGluR5 in the mPFC is expressed mostly on postsynaptic elements [129] to exert excitatory effects on pyramidal cells [130–132]. In contrast, GABAergic inputs to mGluR5 expressing mPFC pyramidal cells are regulated by cannabinoid CB1 receptors under normal conditions [131, 133]. In brain slices from arthritic rats, IPSCs evoked by electrical or optogenetic activation of BLA axon terminals in pre- and infralimbic mPFC pyramidal cells were increased; IPSCs could be blocked with non-NMDA glutamate receptor and GABA_A_ receptor antagonists [71, 126]. Systems electrophysiology studies in anesthetized rats showed that pain-related decreases in background and evoked activity of prelimbic mPFC pyramidal-like neurons were reversed by a GABA_A_ receptor antagonist and attenuated by an mGluR1 but not mGluR5 antagonist [122]. The decrease in mPFC pyramidal cell activity was causally linked to increased BLA neuronal activity in the arthritis pain model because restoring BLA activity with a CRF1 antagonist increased background and evoked activity of prelimbic mPFC neurons [71].

## 5. Pharmacological Strategies Targeting Amygdala Pain Neurocircuitry

Interventions that increase amygdala output, even in the absence of acute injury, elicit pain behaviors [27, 30, 102, 134, 135], whereas those that decrease amygdala activity generally inhibit pain behaviors (see [7] for review). Therefore, controlling amygdala activity is a desirable therapeutic strategy for chronic pain. Interventions that were found to have some beneficial effect in preclinical studies include non-NMDA and NMDA receptor antagonists, mGluR1 and mGluR5 antagonists, agonists for group II mGluR2/3 and group III mGluR, including mGluR8, antagonists for CGRP1 and CRF1 receptors, neuropeptide S activating NPS receptors, and inhibitors of ERK and PKA (reviewed in [7]). Here, we will discuss strategies to control amygdala activity by restoring cortical control as well as interventions targeting the amygdala that have emerged from recent studies.

### 5.1. Strategies Targeting Pain-Related Corticoamygdala Dysfunction

There is good evidence to suggest that mPFC deactivation in pain results in loss of amygdala control (see the Pain-Related Amygdala-Centered Corticolimbic Interactions section). A *CRF1 receptor antagonist* (NBI27914) inhibited the pain-related increase in synaptic excitation and background and evoked activity of BLA neurons in arthritic rats and increased the background and evoked activity of mPFC neurons that was decreased in the pain model [71]. This intervention also inhibited increased mechanosensitivity (spinal withdrawal reflexes), averse affective responses (audible and ultrasonic vocalizations), and anxiety-like behaviors (measured in the elevated plus maze) and restored normal decision-making on a rodent gambling task in arthritic rats [71].

Another strategy to restore mPFC output used a *group II mGluR antagonist* (LY341495) to increase synaptically evoked spiking of mPFC pyramidal cells in brain slices from normal and arthritic rats [136]. Effects of a group II agonist (LY379268) showed that these receptors act on glutamatergic synapses from BLA to inhibit direct excitatory transmission and feedforward inhibition onto pyramidal cells, but their net effect is decreased pyramidal cell output, possibly because the effect on EPSCs preceded that on IPSCs. Facilitatory effects of the antagonist suggest that the system may be tonically active to control pyramidal output.

Activation of mGluR5 was tested because of its location on mPFC pyramidal cells (see the Pain-Related Amygdala-Centered Corticolimbic Interactions section). A *positive allosteric modulator (PAM) of mGluR5* (VU0360172) increased synaptically evoked spiking in mPFC pyramidal cells using electrical and optogenetic stimulation of BLA inputs [126, 131]. This facilitatory effect on mPFC output involved inhibition of synaptic inhibition by engaging endocannabinoid signaling because CB1 antagonists (AM251 and AM281) and an intracellular inhibitor of diacylglycerol lipase DAGL (tetrahydrolipstatin, THL) blocked the effect of VU0360172 [126, 131]. While this strategy worked under normal conditions, the facilitatory effect of VU0360172 was lost in the arthritis pain model due to a breakdown of mGluR5-driven endocannabinoid signaling in the mPFC resulting in a lack of 2-arachidonoylglycerol (2-AG) [126]. The facilitatory effect of mGluR5 activation on mPFC output was restored with inhibitors of the postsynaptic 2-AG hydrolyzing enzyme ABHD6 (intracellular WWL70) and the monoacylglycerol lipase MGL (JZL184) to increase availability of 2-AG in the postsynaptic cell or with a GABA_A_ receptor blocker (intracellular picrotoxin) [126]. Coapplication of *a CB1 receptor agonist (ACEA) with the mGluR5 PAM* also increased synaptically evoked spiking of mPFC pyramidal cell neurons in brain slices from arthritic rats by decreasing abnormally enhanced feedforward inhibition from the BLA through depolarization-induced suppression of synaptic inhibition [126]. Systems electrophysiology studies in anesthetized rats with arthritis showed that coadministration of VU0360172 and ACEA into the mPFC increased background and evoked activity of pyramidal-like cells in the mPFC and inhibited the pain-related increase of background and evoked activity in amygdala (CeLC) neurons [123]. This combination strategy also inhibited increased mechanosensitivity (spinal withdrawal reflexes) and audible and ultrasonic vocalizations and mitigated cognitive deficits in the reward-based decision-making in a rodent gambling task in the arthritis pain model [126]. The data further confirm the inverse link between mPFC and amygdala activity and that restoring mPFC output with a combination strategy of mGluR5-CB1 activation can engage cortical control of abnormally enhanced amygdala output to inhibit pain behaviors.

*Neuropeptide S (NPS)* binds to the G_q_/G_s_-coupled NPS receptor (NPSR), which is expressed in several brain regions including the dorsomedial ITC cell cluster in the amygdala, and produces anxiolytic effects [84, 137–141]. NPS increased mPFC-driven feedforward inhibition of CeLC neurons by activating ITC cell drive and output in brain slices from arthritic rats through a PKA-dependent mechanism [39]. Intra-ITC as well as nasal application of NPS resulted in decreased background and evoked activity of CeLC neurons in anesthetized rats with arthritis pain, and this effect was blocked by stereotaxic administration of an NPSR antagonist ([D-Cys(^*t*^Bu)^5^]NPS or SHA68) into the ITC area [142]. Intra-ITC or nasal application of NPS also inhibited pain-related increases in audible and ultrasonic vocalizations as well as anxiety-like behaviors on the elevated plus maze but had no effect on mechanosensitivity; the inhibitory effects were blocked by stereotaxic administration of [D-Cys(^*t*^Bu)^5^]NPS or SHA68 [39, 142].

These studies provide strong evidence for the concept that engaging mPFC control of amygdala processing may be a useful therapeutic strategy for pain management. This concept is supported by studies in humans that have implicated corticolimbic loops, including mPFC-amygdala interactions, in the prediction of and transitioning to chronic pain [11, 143].

### 5.2. Therapeutic Strategies Targeting Pain-Related Amygdala Hyperactivity

Pharmacological interventions targeting glutamate receptors and neuropeptide systems in the amygdala have been reviewed recently [7]. Here, additional strategies involving the *serotonergic system and potassium channels* will be discussed.

Serotonergic descending pathways are involved in endogenous antinociceptive signaling from the brain to the spinal cord [52, 144, 145]. However, serotonin (5-HT) actions can be excitatory or inhibitory depending on the specific receptor subtype and its associated neurotransmitter [52]. It is therefore not surprising that selective serotonin reuptake inhibitors (SSRIs) have shown inconsistent efficacy for neuropathic pain treatment [146–148]. One of the at least 14 5-HT receptors, the G_q/11_-coupled 5-HT_2C_ receptor, has been implicated in adverse and inconsistent effects of SSRIs for neuropathic pain [149, 150] and, specifically in the BLA, in the generation of anxiogenic behaviors [151–153].

In a rat model of neuropathic pain, viral vector-mediated *5-HT*_*2C*_*receptor knockdown* in the BLA inhibited mechanical hypersensitivity, aversive affective pain behaviors (vocalizations), anxiety-like behaviors, and depression-like behaviors [44]. Pharmacological blockade of 5-HT_2C_ receptors (SB242084) in the BLA conveyed efficacy to a systemically applied SSRI (fluvoxamine) for inhibition of emotional responses (vocalizations) and anxiety-like pain behaviors but not mechanical hypersensitivity [154]. The beneficial behavioral effects of 5-HT_2C_ receptor knockdown in the BLA involved inhibition of irregular and burst firing and evoked activity of CeLC neurons in neuropathic rats [44]. At the synaptic level, 5-HT_2C_ receptor knockdown in the BLA blocked the increase in excitatory transmission at the BLA-CeLC synapse in brain slices from neuropathic rats but had similar inhibitory effects on feedforward inhibition under control conditions and in the neuropathic pain model. 5-HT_2C_ receptor is predominantly expressed in GABAergic neurons, but increased expression in non-GABAergic BLA cells was detected in the neuropathic pain state. The underlying mechanisms of this switch remain to be determined.

Another recent strategy to mitigate pain-related amygdala hyperactivity is *activation of small-conductance calcium-activated potassium (SK) channels* in the CeA. SK channels are calcium-sensitive, voltage-insensitive potassium channels that are expressed in somatic and dendritic regions of the neuron in a brain region-specific manner [155–158]. Somatically expressed SK channels regulate neuronal excitability by mediating the medium afterhyperpolarization (mAHP) to decrease action potential firing rate [155]. In the amygdala, SK channels regulate action potential firing of neurons in the lateral CeA [159] but not LA [160]. SK channels also regulate dendritic excitability to modulate synaptic transmission and plasticity. In the amygdala, activation of synaptic SK channels in the LA acts as a postsynaptic shunt to reduce excitatory synaptic transmission [161], whereas removal of SK channels from the postsynaptic membrane of LA neurons by a PKA-dependent mechanism facilitates excitatory transmission and synaptic plasticity [162].

A clinically available compound that can inhibit SK channels is *riluzole*, an FDA approved drug for the treatment of amyotrophic lateral sclerosis (ALS) that easily crosses the blood-brain barrier [163, 164]. It should be noted that other actions of riluzole include inhibition of voltage-gated calcium channels, rapidly inactivating voltage-gated and persistent sodium channels, and glutamate receptor currents [165–167]. Systemically applied riluzole had antinociceptive effects in the formalin test [168–170], in the carrageenan model of hindpaw inflammation [171], and in neuropathic pain models [172–175]. Riluzole also produced pain relief in patients with irritable bowel syndrome [176]. The site and mechanism of pain-related riluzole effects were not identified in these studies. Systemic application of riluzole inhibited emotional responses (audible and ultrasonic vocalizations), but not mechanosensitivity (spinal withdrawal reflexes), in a rodent model of arthritic pain, and these inhibitory effects were reversed by stereotaxic (intra-CeA) administration of a blocker of SK channels (apamin) but not of large-conductance calcium-activated potassium BK channels (charybdotoxin) [177].

An interesting observation is that not every intervention targeting the amygdala to inhibit emotional-affective responses to pain affects mechanosensitivity. This is true for riluzole [177] as well as for NPS [142], an mGluR5 antagonist [178], and an SSRI [154], and may suggest differential roles of neurochemically distinct intra-amygdala circuits.

## 8. Conclusions

The amygdala is a key node in the interaction of emotional-affective factors with sensory and cognitive aspects of pain. The synaptic and cellular analysis of amygdala function and plasticity as the neurobiological basis of certain pain behaviors has provided a model system for the study of brain mechanisms of pain. The better understanding of relevant intra- and extra-amygdalar circuits and their neurochemical and molecular signatures should yield novel targets for therapeutic interventions because amygdala activity is causally linked to pain behaviors, and therefore, controlling abnormally enhanced amygdala activity is a desirable goal for pain management.

## Abbreviations

2-AG: 2-arachidonoylglycerol
5-HT: Serotonin
ACC: Anterior cingulate cortex
CeA: Central nucleus of the amygdala
CeLC: Laterocapsular division of the CeA
CeM: Medial division of the CeA
CGRP: Calcitonin gene-related peptide
CRF: Corticotropin-releasing factor
EPSC: Excitatory postsynaptic current
ERK: Extracellular signal-regulated kinase
HT: Hypothalamus
IBS: Irritable bowel syndrome
IPSP/C: Inhibitory postsynaptic potential/current
ITC: Intercalated cell mass of the amygdala
LA/BLA: Lateral/basolateral amygdala nuclei
mGluR: Metabotropic glutamate receptor
mPFC: Medial prefrontal cortex
NMDA: *N*-methyl-D-aspartate
NPS: Neuropeptide S
NPSR: NPS receptor
PAG: Periaqueductal gray
PAM: Positive allosteric modulator
PB: Parabrachial nucleus
PKA: Protein kinase A
PKCδ: Protein kinase C delta
SK: Small-conductance calcium-activated potassium channel
SSRI: Selective serotonin reuptake inhibitor.
